# Stroke Treatment Associated with Rehabilitation Therapy and Transcranial DC Stimulation (START-tDCS): a study protocol for a randomized controlled trial

**DOI:** 10.1186/s13063-016-1186-7

**Published:** 2016-01-28

**Authors:** Suellen M. Andrade, Natanael A. Santos, Bernardino Fernández-Calvo, Paulo S. Boggio, Eliane A. Oliveira, José J. Ferreira, Amanda Sobreira, Felipe Morgan, Germana Medeiros, Gyovanna S. Cavalcanti, Ingrid D. Gadelha, Jader Duarte, Joercia Marrocos, Michele A. Silva, Thatiana Rufino, Sanmy R. Nóbrega

**Affiliations:** Cognitive Neuroscience and Behavior Program, Federal University of Paraíba, João Pessoa, Brazil; Perception, Neurosciences and Behavior Laboratory, Federal University of Paraíba, João Pessoa, Brazil; Department of Psychology, Federal University of Paraíba, João Pessoa, Brazil; Cognitive Neuroscience Laboratory and Developmental Disorders Program, Mackenzie Presbyterian University, São Paulo, Brazil; Center for Research in Human Movement Sciences, Federal University of Paraíba, João Pessoa, Brazil; Study Group of Human Movement, Federal University of Paraíba, João Pessoa, Brazil; Neuromuscular Adaptations Laboratory, Federal University of São Carlos, São Carlos, Brazil

**Keywords:** Transcranial direct current stimulation, Stroke, Rehabilitation, Clinical trial

## Abstract

**Background:**

Traditional treatment for motor impairment after stroke includes medication and physical rehabilitation. The transcranial direct current stimulation associated with a standard physical therapy program may be an effective therapeutic alternative for these patients.

**Methods:**

This study is a sham-controlled, double-blind, randomized clinical trial aiming to evaluate the efficacy of transcranial direct current stimulation in activities of daily living and motor function post subacute stroke. In total there will be 40 patients enrolled, diagnosed with subacute, ischemic, unilateral, non-recurring stroke. Participants will be randomized to two groups, one with active stimulation and the other with a placebo current. Patients and investigators will be blinded. Everyone will receive systematic physical therapy, based on constraint-induced movement therapy. The intervention will be applied for 10 consecutive days. Patients will undergo three functional assessments: at baseline, week 2, and week 4. Neuropsychological tests will be performed at baseline and week 4. Adverse effects will be computed at each session. On completion of the baseline measures, randomization will be conducted using random permuted blocks. The randomization will be concealed until group allocation.

**Discussion:**

This study will investigate the combined effects of transcranial direct current stimulation and physical therapy on functional improvement after stroke. We tested whether the combination of these treatments is more effective than physical therapy alone when administered in the early stages after stroke.

**Trial registration:**

NCT02156635 - May 30, 2014. Randomization is ongoing (40 participants randomized as of the end of December 2015).

## Background

A stroke is defined as an acute neurological dysfunction of vascular origin, with sudden development of clinical signs of brain function disorders, lasting more than 24 h [1].

In this sense, new therapeutic modalities have been developed for monitoring patients after a stroke [2]. Simis et al. [3] conducted a placebo-controlled clinical trial and found that transcranial magnetic stimulation (TMS) and transcranial direct current stimulation (tDCS) can cause increased hand motor function compared to placebo stimulation. TMS has been used to minimize the limitations post stroke, such as functional independence and motor recovery [4, 5], but it is not portable/mobile and is expensive. In contrast, tDCS offers some advantages compared to TMS, being portable, more economical, and easy to operate. The effects are polarity-dependent, leading to an increase or a decrease in cortical excitability [6]. Although some studies have shown that increasing the current intensity is related to more robust effects [7], this is also true for adverse effects such as headache and discomfort under the electrode [8]. Therefore, the maximum current intensity used is generally 2 mA, and the cortex density varies between 0.029 and 0.08 mA/cm^2^ [9]. Animal studies with a higher current density of 25 mA/cm^2^ did not induce lesions in the brain tissue, meaning that limits well above those applied in humans did not result in potential adverse effects [10], thereby demonstrating that it is a safe technique.

There is evidence that repeated sessions of tDCS may be associated with a longer duration of the behavioral effects [11]. Monte-Silva et al. [12] demonstrated that the interval between the sessions can be critical to performance. The authors found that when an extra session of tDCS is applied for 1 hour after the first session, the effects last for a longer time (120 minutes) compared to the effect of only one or two consecutive sessions, while an extra session of tDCS applied beyond that period (that is, 3 hours) did not influence the effect of the first session. These findings show that studies with the aim of achieving lasting effects should consider the timing-dependent plasticity stimulation regulation in the human motor cortex [13].

Regarding physical therapy, different approaches can be found for motor recovery, such as mirror therapy [14], repetitive task practice [15], and robotic training [16]. However, the type of training that is combined with stimulation determines how generalizable the benefits would be. Improvements are specific for tasks that are strategically paired with stimulation [17].

In this perspective, efforts are currently being made to standardize the application of the methods that can be combined with tDCS for the treatment of stroke. Bolognini et al. [18] developed a placebo-controlled trial to investigate the neuropsychological and behavioral effects of bihemispheric tDCS (cathodic stimulation in the unaffected hemisphere and anode in the affected cortex) combined with a standard physical therapy program called constraint-induced movement therapy (CIMT) [19]. The data show that CIMT applied alone only seems to be effective in modulating cortical excitability, but is not able to restore the balance of transcallosal inhibition. According to the authors, bihemispheric tDCS can already achieve this goal and promote greater functional recovery. Studies show that CIMT is associated with functional improvement in acute and subacute stages of stroke [20–23]. Although most studies in neurostimulation therapy involve post-stroke patient monitoring for short periods [24, 25], longitudinal studies would clarify the action mechanisms and the effective duration of this association (tDCS plus CIMT) from the early stages of stroke.

The effectiveness of stroke interventions is often described by measures of disability, or functional assessment. Evaluations that deal with activities of daily living (ADLs) generally include the Functional Independence Measure, the Katz index and the Barthel index (BI), the latter being a prevalent measure for the clinical evaluation of stroke patients, with substantial supporting research [26–28]. However, there are few studies involving the ADLs as the primary outcome for a marker of functional recovery after neurostimulation. For example, in a systematic review where the efficacy of tDCS in ADLs and motor function after stroke were analyzed, the authors found that the results are inaccurate and the effect was not sustained when studies of high methodological quality were included. There were 15 studies involving a total of 455 participants included, with only randomized controlled trials and randomized controlled cross-over trials evaluated. Of the total, the analysis of five studies involving 286 participants to examine the effects of tDCS on our primary outcome (ADLs evaluated by BI) has shown that no effect was observed on the performance at the end of the intervention. In three studies from this systematic review involving 99 participants to evaluate the effects of tDCS in BI scores at the end of follow-up, evidence suggested an effect on the ADL performance, but the confidence intervals were wide, and the effect was not sustained when they only included studies with low risk of bias. Thus, the authors point to the need for future research in this area to improve the generalization of the results [29].

Although clinical trials can be found that measure the efficacy of tDCS in ADLs pointing to positive effects [30, 31] among other factors, in general they only include participants in the chronic stage with brain injuries in different areas and varying levels of functional incapacity.

Therefore, central questions remain: For a daily protocol of 10 days, does the active tDCS applied under a 2 mA current and associated with CIMT have a superior response to the simulated (placebo) current applied with CIMT, and if so, what is the size of the effect? What adverse effects are associated with the therapy? Does functional improvement in the ADLs persist over time?

In light of this, a clinical trial phase II/III will be developed to evaluate the therapeutic effects of tDCS in patients in the subacute stage after stroke. The purposes are two: 1) discuss topics related to safety, adverse effects, feasibility, and effectiveness of tDCS in the treatment of stroke patients; 2) present the work protocol prior to clinical trial results, ensuring adherence to protocol. Our hypothesis is that the active stimulation in the affected hemisphere is more effective than a simulated (placebo) current in activities of daily living in subacute stroke. Secondly, we are interested in knowing whether tDCS is effective in the recovery of the following motor variables: spasticity, use of the affected limb, balance, posture, fall risk, muscle strength, and upper and lower limb function. Also, we aim to analyze if a possible functional improvement produces a change in the patients’ perception of their quality of life. We hope that the study will contribute to the discussion of the methodological procedures of clinical trials phase II/III involving neuromodulation for the treatment of patients after stroke.

## Methods

### Overview

**Fig. 1:**
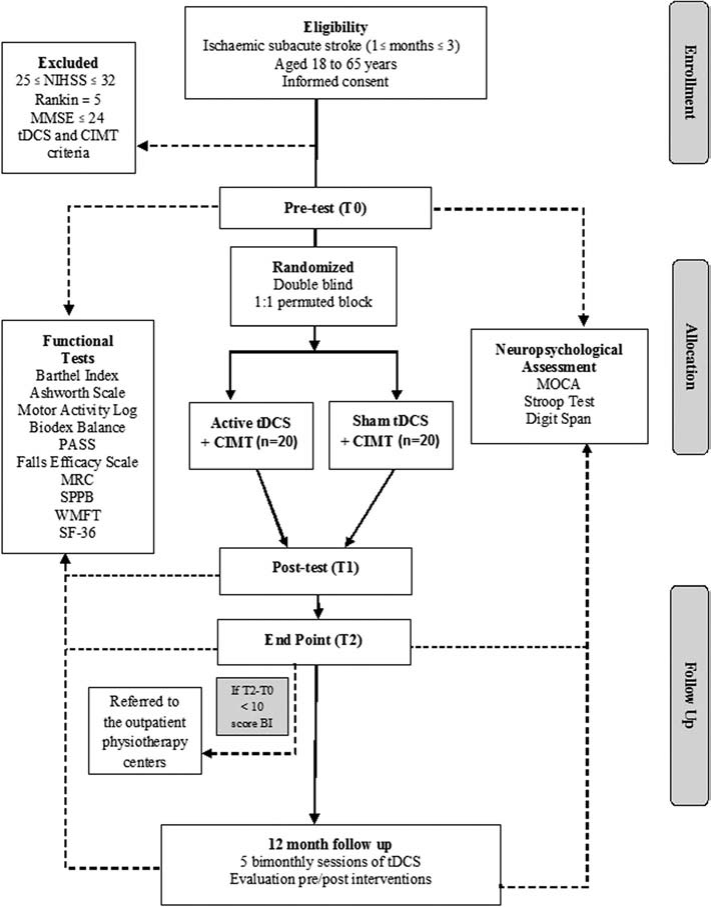
CONSORT (Consolidated Standards of Reporting Trials) flowchart of the clinical trial. BI: Barthel index; CIMT: constraint-induced movement therapy; MMSE, Mini Mental State Examination; MoCA: Montreal Cognitive Assessment; MRC: Medical Research Council (scale); NIHSS, National Institutes of Health Stroke Scale; PASS: Postural Assessment Scale for Stroke; SF-36: Medical Outcomes Study 36-item Short-Form Health Survey; SPPB: Short Physical Performance Battery; tDCS, transcranial direct current stimulation; WMFT: Wolf Motor Function Test

This is a placebo-controlled, double-blind, randomized clinical trial (Fig. 1), in which 40 patients are divided into two groups of 20 participants each. In Fig. 1, the CONSORT (Consolidated Standards of Reporting Trial) flowchart [32] shows the number and distribution of participants: Group A1 - participants in post-stroke subacute stage that receive active tDCS combined with CIMT; Group A2 - participants in the post-stroke subacute stage who receive a placebo stimulation associated with CIMT. Patients will undergo three assessments: baseline (T0), week 2 (T1), and week 4 (T2). Neuropsychological tests will be performed at T0 and T2. Adverse events will be measured in each session, where the researcher responsible for administering the neurostimulation will question the patient on whether they experience any uncomfortable or painful sensation, and if this is related to the tDCS, following the procedure of Brunoni et al. [33]. At the end of the study, participants who received tDCS and showed clinical improvement will be invited to receive bimonthly stimulation for 12 months as part of a longitudinal study of tDCS for stroke. Those who received the tDCS placebo and did not respond will be invited to receive daily sessions with an active current for 10 days. Finally, those who had a response to the placebo will be referred for other physical therapy treatment.

### Patients and enrollment

Participants will undergo neurological, neuropsychological, and physical therapy evaluation performed by specialists in each area as the eligibility criteria for selection.

### Inclusion criteria

Patients will be selected according to the following inclusion criteria: a) age between 18 and 65 years; b) diagnosis of unilateral, non-recurring, subacute stroke of ischemic and lacunar type, as defined by the International Classification of Diseases (ICD-10) through computed tomography or magnetic resonance imaging conducted by neurologists; c) a score < 1 on the consciousness and communication items of the National Institutes of Health Stroke Scale (NIHSS) [34]; and d) the patient provided written informed consent.

Although the subacute phase of stroke generally comprises the period of 7 to 90 days after injury [35–38], this study will only include those at 1–3 months after stroke to avoid the inclusion of participants in the acute phase. The definition of the age group followed the age interval often found in studies involving tDCS and stroke [17, 39, 40]. However, for a more conservative position of experimental control, we preferred to limit the inclusion up to 65 years of age to avoid this variable possibly interfering in the performance of elderly participants, as this could increase the chances that there are other conditions associated with aging (such as concentration and memory).

### Exclusion criteria

Patients will be excluded if they have: a) a score between 25–32 points on the NIHSS [34] and grade/degree 5, according to the Rankin scale [41]; b) cognitive deficits (a score below 24 points on the Mini Mental State Examination) [42]; c) an inability to actively carry out wrist flexing/bending, that is, metacarpophalangeal and interphalangeal active extension of 10° to 20° wrist extension.

In addition, exclusion criteria relating to contraindications of neurostimulation will be applied, following the safety guidelines [9, 13]: a) use of psychoactive drugs, as stated in the recommendations; b) patients with implanted metallic or electronic devices; c) cardiac pacemaker; d) seizures; e) acute eczema under the electrodes, the presence of tumors, epilepsy or substance abuse; f) pregnancy (this condition was specifically enlisted as a contraindication in this study as a precaution, as there are no data on maternal and fetal effects of tDCS on pregnant women who have suffered a stroke).

### Recruitment

A sample of volunteer participants will be recruited from hospitals and rehabilitation clinics, linked to the local public health system, as well as by study information provided on websites, newspapers, and radio. There will also be referred local support groups, outpatient, and community programs aimed at stroke treatment.

In addition, health professionals from the referred locations where this study will be performed (hospitals and rehabilitation clinics) will disseminate the research among their patients who have suffered stroke. After prior written consent, patients interested in participating will have their records analyzed and will be contacted for inclusion in the study, according to the eligibility criteria. Thus, medical records will only be accessed with prior consent, avoiding possible bias and disregard for privacy legislation, as pointed out by Kho et al. [43].

### Ethics, study registration, and participant consent

The study is being conducted in accordance with the principles of the Helsinki Declaration [44] and the guidelines of Good Clinical Practice (GCP) of the International Conference on Harmonization of Technical Requirements for Registration of Pharmaceuticals for Human Use (ICH) [45]. The study was approved by the local Ethics Committee of the University of Paraíba (number 30163714.0.0000.5188; João Pessoa, Brazil), and is registered in the ClinicalTrials.gov database (NCT02156635). We followed the SPIRIT (Standard Protocol Items: Recommendations for Interventional Trials) guidelines for writing clinical trial protocols [46].

All participating patients will give written informed consent. During the consent, the researcher clarifies the objectives and the procedures to be used in research, with details of the methods to be used, the risks and benefits, and stating the possibility of inclusion in a control or experimental group. The consent also provides a full guarantee of freedom for the participant to refuse to participate or withdraw their consent at any stage of the research, without any penalty, and shall maintain the confidentiality and privacy of the participants during all phases of the research. All participants will receive a copy of the consent form approved by the responsible Ethics Committee. To guarantee that all details of the study process were understood, the researcher has formulated open questions to ask participants on the content of the consent, and only those who answer correctly and sign the document may enter the study.

The study results will be presented at conferences and published in international peer-reviewed journals regardless of the direction and magnitude of the effect.

### Outcomes

For analysis of the primary endpoint, the Barthel index (BI) was chosen because it is commonly used in studies related to post-stroke rehabilitation, adequately reflecting clinical practice [47–49]. This instrument is designed to assess the patient’s level of independence to perform ten basic life activities: eating, bathing, personal care, ability to dress themselves, motility, urinary regularity, bathroom use, chair-bed transfer and vice versa, mobility, and climbing stairs [50]. The literature on the minimal clinically important difference detected by the BI shows that a change of 2 points is meaningful and beyond measurement error. This calculation refers to a modified BI with scores of 0–20 points [51]. Since the changes in the values of the scores do not affect other properties of the scale, it is considered equivalent to the original version, with scores of 0–100 points [52]. Thus, taking the original scale used in this study as a reference, clinical improvement will be considered as a final score higher than 10 points (T2) according to the BI, compared to baseline (T0) before the therapeutic protocol [30].

The BI has been reported with excellent levels of reliability, validity, and adequate responsiveness to change in several countries [52, 53]. In a study of 121 patients at four post-stroke period points (14, 30, 90, and 180 days), the inter-rater reliability using a weighted kappa varied from 0.53 (adequate) to 0.94 (excellent). The inter-rater reliability for the total score, calculated using the intraclass correlation, was 0.94 (excellent). The internal consistency, calculated using Cronbach’s alpha, was excellent, varying between 0.89–0.92 [54]. The construct validity was also confirmed by Wilkinson et al. [55], with rank correlation coefficients of the BI with the SF-36 (Medical Outcomes Study 36-item Short-Form Health Survey) subscales being found in patients with their first-ever stroke varying from r = 0.22 (Role Emotional subscale) to 0.81 (Physical Functioning Profile subscale).

Secondary outcome measures include the Modified Ashworth Scale, the 30-item Motor Activity Log, the Biodex Balance electronic platform, the Postural Assessment Scale for Stroke, the Falls Efficacy Scale, the Medical Research Council scale, the Short Physical Performance Battery, the Wolf Motor Function Test, and the SF-36.

The Modified Ashworth Scale, used in semiotic clinics for spasticity evaluation, evaluates the tone in grades of 0-4 points, and is widely used in clinical practice with adequate test-retest and inter-rater reliability [56–58].

The 30-item Motor Activity Log measures the spontaneous use of the affected limb. Each item asks about the frequency and quality of movement in daily activities of the paretic upper limb. Scores on this measure have adequate reliability and validity in individuals with stroke [59, 60].

The Biodex Balance electronic platform measures the static and dynamic balance through different levels of stability, such as anteroposterior and mediolateral. This instrument is used in several studies involving tDCS applied to stroke [61, 62].

The Postural Assessment Scale for Stroke (PASS) for postural control [63] contains 12 items with progressive levels of difficulty, involving three fundamental postures (lying, sitting, and standing).

The Falls Efficacy Scale measures the fear of falling in 16 ADLs. The total score can vary from 16 (no concern) to 64 (extreme concern) [64, 65].

The force scale from the Medical Research Council (MRC) consists of manual muscle testing, varying from 0 (no movement is observed) to 5 (normal force against the total resistance), with a maximum score of 60 points [66].

The Short Physical Performance Battery (SPPB) consists of three tests that evaluate the static standing balance, gait speed in normal step, and, indirectly, the muscle strength of the lower limbs. Each domain varies from 0 to 4 points, and the maximum total score indicates better physical performance [67, 68]. Gait velocity is a relevant indicator of function and prognosis after stroke [69], meaning that the SPPB is an important tool for assessment of lower extremity motor recovery [70–72].

The Wolf Motor Function Test (WMFT) assesses the speed of task execution through time, the quality of movement, and the strength of grip and shoulder flexion in specific tasks. The WMFT tasks should be filmed with a camera placed at standard position and distance, and the score of the tasks are given from video analyzes [73, 74].

The SF-36, a multidimensional questionnaire to analyze the state of health, consists of 36 items encompassing 8 components, with a final score of 0 (worst state) to 100 (best state) [75].

### Randomization and blinding

The method of randomization will be a 1:1 permuted block randomization generated by a web-based randomization tool (www.random.org). This sequence will be done independently and remotely by a blinded investigator who will not have contact with other research procedures. The randomization will be concealed until group allocation.

After the randomization process, a blind researcher not involved with the recruitment, data collection, or intervention will perform the allocation of participants between the groups. This will be employed by concealed allocation of sequentially numbered, opaque, sealed envelopes, so that the person responsible for allocation will not have contact with patients or with the work of others. This envelope will be delivered one day before the treatment sessions to the researcher responsible for neurostimulation, who will not be involved with the other procedures for collection or data analysis.

Participants will not be identified by their real names and will not be aware of which arm of the study they are allocated to. When included in the study, they will receive a sealed, opaque envelope containing their respective identification code. In this way, the assessors will also be blind, as they will be identifying the patients by codes and will not have contact with other research procedures. The same will happen to the staff responsible for the execution of CIMT procedures. Data analysis will be conducted by a researcher not involved in any stage of recruitment, screening, assessment, or intervention.

While evaluating the efficiency of the masking mechanism [17], which is routine in studies for the treatment of stroke and other diseases related to neuromodulation, biases may occur due to inadequate blinding of several people involved in the clinical trial, or by error allocation, the effect of the treatment, or co-interventions [76]. Unlike blindness, these bias-generating consequences can be measured [77]. To this end, the example of another study using external judges [78] will be applied in this work, a final evaluation involving neurologist doctors accompanying the patients in their clinical treatment. These professionals will not be aware of behavior and interventions applied in this experiment. The imaging tests carried out for the diagnosis of vascular injury will be repeated and evaluated by these doctors at the end of intervention (T1) as a routine consultation procedure. After analysis of these imaging tests, the doctors will be asked whether they believe that the patient has suffered some consequential event which can act as an external assessment tool for a clinical trial. Reports of biased results can be examined as to whether the degree of agreement between the opinion of an external judge (neurologist) and the trialists (involved in outcome assessments and physical therapy interventions with CIMT) will be the same for both experimental and control participants.

## Intervention

### Constraint-induced movement therapy (CIMT)

The CIMT will be held immediately after the patient has received a neurostimulation session. Daily 3-hour protocol motor skills training supervised by a therapist will be applied for two weeks (10 calendar days, excluding weekends), with non-affected limb restriction for 90 % of the patient’s waking time, as advocated in previous studies with patients after stroke [79]. The motor training conducted by a physiotherapist will cover the use of the paretic limb during ADLs, with the non-paretic arm restricted by a sling. The patient will be encouraged to use the affected limb during the daily routine of his/her activities.

Patients will be encouraged to keep a journal to document their workouts, recording the training time duration, activities done with the restriction sleeve, and main difficulties. The physiotherapist responsible for the treatment will review the diary weekly, which will allow for controlling and verifying compliance in the implementation of CIMT performed by the patient. If there is a discrepancy with the principles of the technique, adjustments and guidelines will be offered for the proper conduct of the protocol. It is important to clarify that the purpose of the restriction sleeve is not to encourage a permanent change in the way the patient performs daily activities, but to encourage the concentrated and repetitive use of the paretic upper limb, seeking a “use-dependent” cortical reorganization [80].

Thus, all participants, regardless of receiving active or simulated current, will be treated with CIMT during the 10 days of neurostimulation, respecting the ethical principles to ensure therapeutic assistance to those involved.

### tDCS

The planning of the stimulation variables involves intensity, frequency of sessions, size/position of the electrodes, and duration of treatment [9]. The review of the therapeutic use of tDCS which defined safety variables was based on 21 studies (n = 278), and the conditions included depression, Parkinson’s disease, cerebral ischemia, eating disorders, alcohol dependency, and chronic pain syndromes. The application of an anodic current with identical variables to this study (involving the use of 35 cm^2^ electrodes and applying a current of 2 mA for 20 minutes for 10 days) was not related to long-term adverse or high magnitude effects [81].

The electrodes are positioned on the participant’s head at the primary motor cortex area, C3 or C4 position according to the electroencephalogram 10–20 system [82]. The anode is placed on the affected hemisphere and the cathode (reference electrode) on the supraorbital region in the uninjured hemisphere [83].

The protocol will be for 20 minutes of stimulation for 10 days (excluding weekends). The TCT neurostimulator (Research Version) developed by Trans Cranial Research Limited (Hong Kong, China) will be used, with the kit containing the neurostimulator, sponges, rubber fasteners, electrodes, and connecting cables. Electrodes are encased with 5 × 7 cm sponges and moistened with saline (0.9 % NaCl). The applied current is 2 mA, with a relative current density of 0.05 A/m2.

The protocol is identical for placebo stimulation, but the current will stop after 30 seconds from the start of stimulation, a blinding method considered reliable for several previous studies [9, 84], in which the active current is simulated (slight tingling and itching sensation) for a short period, and the effects disappear shortly after the start of the stimulation.

Although the required number and maximum sessions have not been established, we prefer to follow the model used in previous studies, which for most cases had consecutive sessions of tDCS applied for 10 days, and was able to improve motor function in different phases of stroke [24, 85, 86].

### Attrition and adherence

Attrition will be considered under the following conditions: a) two consecutive or three alternate absences during treatment sessions; b) inability to complete the post-test and follow-up; c) development of any disabling condition for participation in the study. Regarding adherence strategies, up to two non-consecutive absences can be compensated the following week. There will also be flexible hours offered for receiving therapy, as well as direct contact by telephone with participants confirming the evaluation dates and reinforcing treatment adherence [33]. Additional measures to prevent dropouts will also be applied, such as periodic evaluations (during the outcome analyses) on satisfaction with therapy, discussion of difficulties in continuing with treatment (for example, transport logistics to the laboratory), and attempts to resolve and prevent possible problems that may interfere with adherence and continued participation in the study.

### Safety

With regards to adverse effects, patients will be asked at every stimulation session about the effects experienced such as “tingling,” “burning,” “headache,” “sleepiness,” and others, which they will then score in intensity (1 - no, 2 - mild, 3 - moderate, 4- strong), and this effect is related to the stimulation in a Likert scale of 1 (no relation) to 5 (strongly related) [33].

Regarding safety, deleterious cognitive effects, that is any effect from therapy detrimental to cognitive functions, will be analyzed using the following neuropsychological tests referenced in the literature: Montreal Cognitive Assessment (MoCA), which assesses cognitive dysfunction [87]; the Victoria version of the Stroop Color and Word Test, to measure executive functions [88]; the Digit Span subtest, which evaluates attention and (direct order) and working memory (indirect order) [89]. A cognitive evaluation will be conducted at the beginning and end of the study to check if there really are deleterious effects of neurostimulation, so that the values at baseline can be used as a reference.

We could find no literature reports of any deleterious cognitive effect after application of tDCS. It is considered a safe technique that has been used for several years in post-stroke patient treatment [9, 22, 33, 81].

If the patient experiences any loss or strong discomfort, the treatment will be stopped and medical care as well as physical and psychological therapy will be offered for control of potential problems and to promote recovery.

### Data analyses

The design of the statistical analysis is based on previous studies of literature relating to clinical and placebo-controlled trials using tDCS [17, 30, 90, 91]. The intention-to-treat analysis will be used; however, high or differential rates of missing data between treatment arms/sides may indicate a departure from this assumption and may lead to bias [92]. Thus, a sensitivity analysis will be performed with different allocation procedures to verify the strength of the data. The best strategy resulting from the comparison of the following methods will be chosen: last observation carried forward, complete case analysis, likelihood-based methods, and multiple imputation [93]. As pointed out by White et al. [94] in the intention-to-treat analysis strategy, the main focus in the analysis of choice should be the plausibility of its assumptions, while the inclusion of all randomized individuals is a requirement only for sensitivity analysis.

Multivariate imputation by chained equations (MICE, the R Foundation for Statistical Computing, Vienna, Austria) and SPSS Ver.20.0 (SPSS Inc., Chicago, IL, USA) software will be used.

Regarding sociodemographic and clinical characteristics at the baseline, for continuous variables, information on the variability will be presented for example, mean and standard deviation). If groups are similar to each other, descriptive data analysis will be performed. However, if important differences are found, regression analysis will be used to evaluate these data to estimate the effects of covariates with the variables of interest.

Regarding clinical outcomes, the primary outcome will be analyzed with analysis of mean change scores (MCS) to the total score of BI. The MCS will be evaluated in three ways: change occurred between the post and pre-test (T1-T0); between endpoint and pre-test (T2-T0); between the endpoint and post-test (T2-T1). The baseline as covariate will be used to identify potential differences between the groups by analysis of covariance (ANCOVA) [95].

Finally, the effect sizes and confidence intervals of change scores will be calculated using Cohen’s *d*, using the Hedges correction, with values of 0.20, 0.50, and 0.80, reflecting small, medium, and large effects, respectively [96]. For all variables of the secondary outcomes, the same analysis strategy will be used. Non-parametric tests will be performed if some of the outcomes do not meet the conditions necessary for parametric analysis.

Regarding safety, deleterious adverse and cognitive effects will be recorded/computed in terms of the proportion in each group, in each time period (T0, T1, and T2), and will be analyzed by the Fisher exact test.

Bias-generating will be tested. The statistical model used for blinding effectiveness was based on the study of Brunoni et al. [97]. The chi-square test or Fisher’s exact test will be used to assess if tDCS blinding guessing was beyond chance for patients, external judges, and trialists. Paired *t*-tests will be used to compare the rate of their guessing according to the Likert scale. Logistic regressions exploring the factors associated with “correct blinding guessing” (that is, participants who correctly guessed their allocation group) will be performed. The factors will be clinical response, high guessing confidence (scores 4 or 5 on the Likert scale), and adverse effects.

### Sample size

The sample size was estimated based on previous studies using matched groups of non-invasive stimulation with rehabilitation [98, 99]. The power calculations to determine the number of participants in each group were determined in relation to the expected change in BI because it is commonly used in tDCS studies of patients after stroke [30, 31].

According to Khedr et al. [30], the mean expected improvement is about 10 points, with a standard deviation (SD) equivalent to 7 points. Thus, a calculation considering 90 % power suggests that it would take at least 12 patients in each group to detect the difference found corresponding to the effect of active or placebo tDCS.

Considering the possibility of sample loss during the study (withdrawal, inability to continue the treatment, mortality), 20 patients will be included per group, totaling 40 participants (SPSS Ver.20.0; SPSS Inc., Chicago, IL, USA). The analysis will be performed at a 0.05 significance level.

## Discussion

The present work is a placebo-controlled, double-blind, randomized clinical trial developed to analyze the effectiveness of tDCS in activities of daily living of stroke patients. Regarding the sample, the majority of clinical trials involving neuromodulation and rehabilitation involve samples of 20 to 30 subjects and follow-up conducted for approximately three months [17, 100]. The use of a larger sample, with monitoring of the respondents to therapy during a year as proposed in this study reduces the risk of type II error, allowing for the possibility of expanding the generalization spectrum into other contexts related to tDCS.

Furthermore, although other studies have currently been developed on this subject, most of the work has methodological differences, such as lack of control on the type of stroke, lesion location, and characteristics of the participants [91]. Patients with ischemic stroke should not be directly compared with those who have had a hemorrhagic stroke; also, patients with multiple foci and extensive areas of injury should not be equated to those with mild or moderate degrees of compromise. Errors of this nature committed during the inclusion of the participants are methodological limitations, inhibiting confirmation or refutation of raised hypotheses.

In this sense, questions remain about at what moment after stroke tDCS therapy would be appropriate. Elsner et al. [29] developed a systematic review of the application of tDCS on motor function and ADLs, and verified the existence of three studies involving subacute patients, confirming that most of the work involves patients analyzed more than six months after the stroke. Therefore, there is need for controlled clinical trials evaluating the effect of this technique on patients in the early stages of a stroke.

As for the therapies associated with neurostimulation, some studies include patients with tDCS not assisted by any intervention or uniformed treatment protocols [91, 99]. A Cochrane meta-analysis examined studies using physical therapy techniques used in post-stroke rehabilitation. Although the authors agree that physical therapy is a key part of rehabilitation, the study’s findings were not able to measure which technique was more effective than others. In this sense, the authors recommend that future research should focus on investigating the effectiveness of clearly described techniques and standard treatments for the tasks developed [29]. Another systematic review, which analyzed randomized controlled trials on the treatment of various therapies involving upper limbs after stroke, notes that there is a limitation in terms of scientific evidence of the benefits of conventional therapies such as stretching, passive exercises, and mobilization [101]. According to the authors, the methodological flaws involving these procedures do not make it clear how much therapy is to be provided, who should provide it, and how patients should be directed so that the functional gains are maximized.

The lack of control of these techniques with rehabilitation studies can bring forward biases with confounding variables in interpreting the results, beyond what prevents the replication of future studies in the area. Techniques not standardized can be considered inferior to the systematic protocols of physical therapy. Instead, the standard motor training, if coupled with tDCS, can guide the plasticity process towards a functional result, since tDCS activates a mass of circuits which receive neuromuscular feedback in a specific way [17]. Given these facts, standardized and systematic CIMT physical therapy was chosen for this study, taking into account that since the CIMT therapy may promote recovery through inter-hemispheric inhibition, the tDCS can also induce beneficial effects of focal mode, activating neural circuits in the damaged hemisphere [18].

Overall, this study will assess the effectiveness of tDCS combined with CIMT physical therapy protocol in subacute stage after-stroke patients, and analyze the maintenance of the benefits achieved over time through markers of functional recovery. Therefore, the results of this study may lead to implications for future clinical trials in this area.

### Study limitations

Some limitations to our study should be discussed. One is the adherence. Patients will have to get to the research center for 10 consecutive days, and therefore there may be absences due to transportation problems or personal difficulties. However, different strategies will be adopted to minimize these problems, such as flexible schedules and frequent phone contact, encouraging and reinforcing the importance of treatment. Brunoni et al. [33] had a total of 9 losses after three weeks of stimulation and 17 at the end of the study (after six weeks), with application of similar measures in patients with depression. These numbers refer to a dropout rate of 7.5 % from 120 patients analyzed. Second, the proposed design does not include patients with severe functional limitations. This criterion was inserted because patients with extreme degrees of disability often exhibit extensive lesions. In these cases, there is often important impairment of the corticospinal tract, which could limit the patient to be chosen for physical therapy protocol, since there is the requirement of a minimum level of functional ability to perform the tasks. Accordingly, further studies on this population could include tDCS treatment combined with another type of rehabilitation program with adequate systematization, allowing replication of the data found.

## Trial status

The study was registered at ClinicalTrials.gov on 30 May 2014. Recruitment started on 30 December 2014 and will proceed until November 2015. Randomization is ongoing (40 participants randomized as of the end of December 2015). The final report will be prepared for 2016.

## Abbreviations

ADLs: activities of daily living
ANOVA: analysis of variance
BI: Barthel index
CIMT: constraint-induced movement therapy
CONSORT: Consolidated Standards of Reporting Trials
ICD-10: International Classification of Diseases
MCS: mean change score
MoCA: Montreal cognitive assessment
MRC: Medical Council Research (scale)
NIHSS: National Institutes of Health Stroke Scale
PASS: Postural Assessment Scale for Stroke
SF-36: Medical Outcomes Study 36-item Short-Form Health Survey
SPIRIT: Standard Protocol Items: Recommendations for Interventional Trials
SPPB: Short Physical Performance Battery
tDCS: transcranial direct current stimulation
TMS: transcranial magnetic stimulation
WMFT: Wolf Motor Function Test
